# Wild-Edible *Allium* Species from Highlands of Eastern Anatolia: Phytochemical Composition and In Vitro Biological Activities

**DOI:** 10.3390/plants13141949

**Published:** 2024-07-16

**Authors:** Muzaffer Mukemre

**Affiliations:** Department of Plant and Animal Production, Yuksekova Vocational School, Hakkari University, Hakkari 30100, Türkiye; mukemre.muzaffer@gmail.com or muzaffermukemre@hakkari.edu.tr

**Keywords:** *Allium*, allicin, phenolics, biotherapeutic, Eastern Anatolia

## Abstract

This study presents the phytochemical composition, antioxidant (hydrogen atom and single-atom transfer mechanisms), and digestive enzyme inhibitory (alpha-amylase, alpha-glucosidase, and pancreatic lipase) activities of ethanol-based extractions and traditional preparations (infusion) of the leaves of wild-edible *Allium* species (*A. kharputense*, *A. affine*, *A. shirnakiense*, and *A. akaka*) from the highlands of Eastern Anatolia. Among the eight extracts analyzed, ethanol extractions of the *A. kharputense* and *A. akaka* leaves exhibited better biotherapeutic activities and had the highest bioactive content. The dominant bioactive profile was composed of mainly allicin and phenolic compounds (chlorogenic acid, hesperidin, rutin, isoquercitrin, and quercetin) with small amounts of fatty acids. These data were similar to the biological activities and chemical composition of common *Allium* species and suggest the utilization of the extracts of wild-edible *Allium* species in the development of *Allium*-based biotherapeutics or nutraceuticals.

## 1. Introduction

Plant-based natural materials are extensively used as food in Eastern Anatolia, which represents a rich traditional knowledge of food plants due to its rich biodiversity, and local flora and fauna. Also, their herbal infusions have been used as local medicines to treat various ailments, such as inflammation, diabetes, stomachache, hemorrhoids, colds, and cancer [1]. Of them, a high number of *Allium* species have been used as food and medicine due to their common vegetation potential in the wild. Ethnobotanical studies revealed the extensive uses of leaves and bulbs of *Allium* species as food and medicine [2,3], and their therapeutic and nutritive potentials were later reported [4,5,6].

It has been reported that more than 600 *Allium* species grow in the wild [7] and only a limited number of these species, including onion (*Allium cepa*) and garlic (*Allium sativum*), has been cultivated and consumed by several ethnic groups across the world [3], which contain significant nutraceuticals and phytochemicals useful for the treatment or management of metabolic disorders [8], cancer, and cardiovascular diseases, and exhibit numerous biological activities, including antimicrobial, antiviral, antioxidant, antispasmodic, antimutagenic, neuroprotective, and immunomodulatory activities [9,10]. However, it was reported that local *Allium* species showed comparable biological activities to cultivated *Allium* species, including onion and garlic [11].

Due to its tribal and semi-nomadic lifestyle, local foods have represented a high scale of plant foods in Eastern Anatolia until now. *Allium* species are not only significant flavoring agents and food bioresources, but also significant sources of medicinal practices in Eastern Anatolia. In addition to commonly cultivated *Allium* species (onion and garlic), a great number of *Allium* species have been used in the preparation of food (herbal cheese, omelets, sauces etc.) and medicine (particularly diabetes) in the region. Of them, *A. kharputense*, *A. affine*, *A. shirnakiense*, and *A. akaka* are commonly collected from the highlands and stored as dried for later use [2,12]. The bulbs and leaves of these species are generally utilized as an infusion for medicine or eaten raw for food. Though they have extensive utilization, scientific reports regarding their biological activities and chemical composition are limited.

Plants can produce many diverse bioactive compounds, such as phenolics, carotenoids, and tocopherols, which have medicinal properties and nutritional value. High concentrations of phytochemicals accumulate in plant organs, which act as natural antioxidants and enzyme inhibitors. To extract phytochemicals from the plant matrix, various extraction methods, including solvent-based, microwave-assisted, enzyme hydrolysis, supercritical fluids, and ultrasonic-assisted methods, are applied. Of them, the solvent-based extraction method has been extensively utilized because of its effectiveness, ease of use, and wide applicability. One of the most important advantages of the solvent-based extraction method is the polarity index of the solvent used, which provides to the extract targeted phytochemicals from the plant matrix [13,14,15]. Therefore, we hypothesized that traditional preparations (infusion) and ethanol-based extracts of these species must be analytically analyzed to understand their chemical nature, as well as reveal the beneficiation percentage in the diet for human health and determine the potential of local *Allium* species as functional foods for the management of enzyme inhibition-related metabolic diseases.

## 2. Results

### 2.1. In Vitro Biotherapeutic Potential

#### 2.1.1. Extraction Yields

The extraction yields of the extracts obtained from the leaves of *Allium* species are presented in Table 1. Among all the extracts analyzed, the *A. affine* and *A. akaka* plant materials gave the highest yields through the infusion method both for leaves. The results showed that the use of water and the application of heat treatment showed a better extraction of phytochemicals from the plant matrix.

#### 2.1.2. Total Phenolic Contents and Antioxidant Activities

Total phenolic contents and antioxidant activities through the single-electron transfer mechanism, which was represented by the FRAP assay, showed that the ethanol extract of *Allium akaka* leaf sample (48 mg gallic acid E and 872 μmol Fe^2+^/g extract, respectively) had the highest results. With regard to the ORAC assay, which represented the hydrogen atom transfer mechanism, ethanol extracts of the *Allium akaka* and *Allium kharputense* leaf samples showed better antioxidant activities. Also, the ethanol solvent had a higher capacity for extracting antioxidant compounds than the water solvent (Table 1). These findings are comparable with several *Allium* species, such as *Allium cepa* [16], *Allium roseum* [17], and *Allium scorodoprasum* [18].

#### 2.1.3. Enzyme Inhibition Activities

The extracts showed moderate inhibitory activities against alpha-glucosidase and pancreatic lipase, but not amylase. However, the extracts were found as weaker inhibitory agents according to the positive controls: acarbose and orlistat. Like antioxidant capacities, the ethanol extracts were stronger than infusion preparations (Table 2). Among all samples tested, *Allium akaka* showed the highest inhibitory activities (IC_50_: 500 and 195 μg/mL for α-glucosidase and pancreatic lipase, respectively). The inhibitory activities of the extracts against α-glucosidase were high and against α-amylase were low, which was like the inhibitory activities of *Allium cepa* extracts [16]. In agreement, Nickavar and Yousefian [19] reported that *Allium* species are weak inhibitors of α-amylase.

#### 2.1.4. Phytochemical Composition

The phytochemical compounds identified using HPLC-PDA-MS/MS are shown in Table 3. Six dominant compounds were identified as described below. The first compound was identified as chlorogenic acid according to a maximum absorption at 326 nm and a charged molecular ion of 353 m/z in the negative ion mode with the detection of a fragment ion of 191 m/z in the SRM mode. This compound was detected at very high levels in all samples, excluding *A. shirnakiense.*

**Figure 1:**
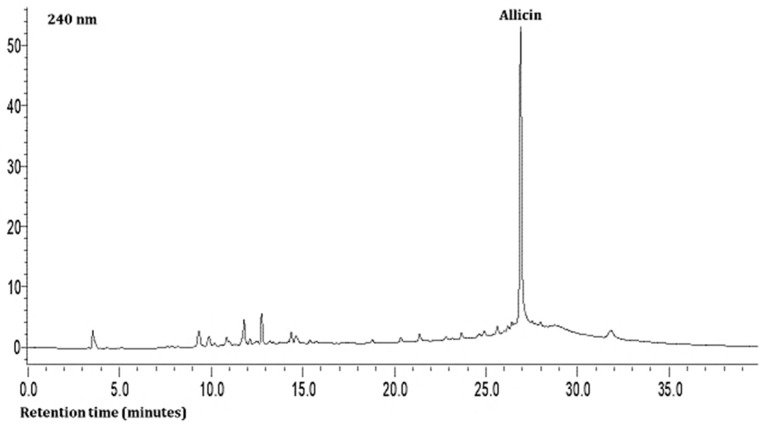
Representative HPLC chromatogram of *Allium* leaves at 240 nm.

The second compound was characterized as hesperidin based on a charged molecular ion of 609 m/z in the negative ion mode with the detection of fragment ions of 449 and 431 m/z in the SRM mode. This was found in all the leaf samples at low levels (Table 3). The third, fourth, and fifth compounds present in the extracts were quercetin and its glycosides (rutin and isoquercitrin) at very high levels based on the MS data. The seventh compound was identified as allicin based on a charged molecular ion of 163 m/z in the positive ion mode with the detection of a fragment ion of 121 m/z in the SRM mode (Table 3; Figure 1). This compound was found in all the samples at high levels. 

**Figure 2:**
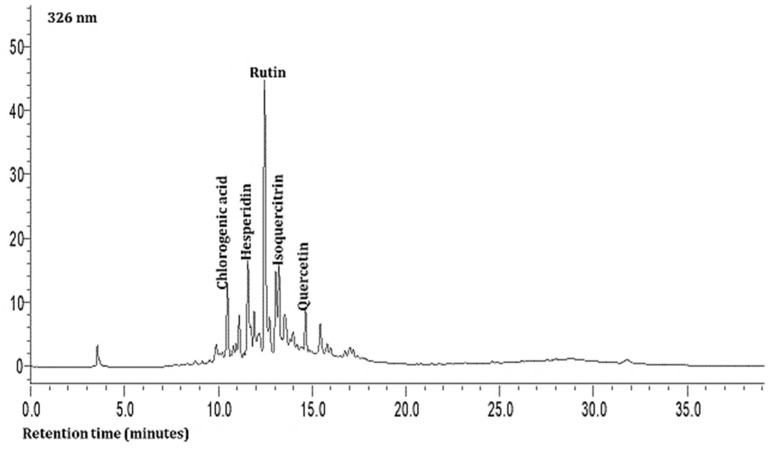
Representative HPLC chromatogram of *Allium* leaves at 326 nm.

As shown in Table 3 and Figure 1 and Figure 2, the hydrophilic composition of the infusions and ethanol-based extracts mainly consisted of allicin (Figure 1), an organosulphur compound, and various phenolic compounds, including chlorogenic acid, rutin, isoquercetin, and quercetin (Figure 2). It was found that the *Allium akaka* and *Allium kharputense* samples suggested rich sources of phenolics. Most phenolic compounds consisted of flavonols with the domination of quercetin and its derivatives. The representative compound of phenolic acid was found as chlorogenic acid.

On the other hand, fatty acids (palmitic, stearic, linoleic, and linolenic acids) dominated the lipophilic composition of the extracts (Table 4). The major fatty acids were found to be oleic, palmitic, and linolenic acids.

## 3. Discussion

The solubility of phytochemicals is governed by the chemical nature of the plant sample, as well as the polarity of the solvents used. The choice of the solvent in the extraction procedure affects the recovery of phytochemicals from the plant matrix. For example, a low polarity-indexed solvent, such as n-hexane or chloroform, can extract fatty acids, waxes, and terpenoids, while a medium polarity-indexed acetone can extract less polar and polar flavonoids, tannins, terpenoids, and glycosides, based on the chemical nature of the plant material extracted [13]. The chromatographic studies revealed that those solvents (water and ethanol) used in this study extracted the same compounds at different concentrations. The ethanol solvent had a higher capability of extracting hesperidin, rutin, isoquercitrin, quercetin, allicin, and chlorogenic acid. Also, it can be suggested that the heating procedure used in the infusion method had no remarkable effect on the extraction of phytochemicals from the plant matrix (Table 3). Kim et al. [16] reported that using ethanol is more convenient to recover phytochemicals, particularly quercetin, from onion skin. Scientific reports showed that the major phytochemicals present in *Allium* species differ according to the plant material studied, such as quercetin in *Allium cepa* [16]; quercetin, luteolin, and eriodictyol in *Allium scorodoprasum* [18]; kaempferol and its glycosides in *Allium tuberosum* [20] and *Allium roseum* [17]; allicin and isoquercitrin in *Allium schoenoprasum*; rutin in *Allium senescens* [21]; rutin, quercetin, and kaempferol in *Allium flavum* [22]; and quercetin 3,4′-diglucoside, quercetin 4′-monoglucoside, quercetin aglycone, isorhamnetin, and kaempferol in *Allium cornutum* [10]. Moreover, Schmidt et al. [11] investigated the phytochemical composition of 35 *Allium* species and found quercetin to be a representative phenolic compound of the extracts, which agrees with our findings. Like our findings, allicin was found in 33 *Allium* accessions representing 14 species, which makes it a taxonomic key compound [23]. Rutin, chlorogenic acid, isoquercitrin, quercetin, and allicin were found to be major phytochemicals of *Allium* species in this study, which agrees with previous studies conducted in common and local *Allium* species [18,21,22,24]. Also, the presence of quercetin and its glycosides, which are among the most common phenolics of wild plants, showed the adaptation abilities of *Allium* species in the wild.

One of the most effective therapeutic approaches to manage postprandial hyperglycemia is to limit the activity of the carbohydrate digestive enzymes involved in the release of glucose from the daily diet. Alpha-amylase and alpha-glucosidase are among the key enzymes in the digestive process that are responsible for converting polysaccharides to disaccharides and monosaccharides, which results in the increasing of the blood glucose level. α-Amylase breaks down large polysaccharides into sugars in the saliva. It is the key enzyme that degrades the polymeric substrate into shorter oligomers by catalyzing the hydrolysis of the α-1,4-glucan linkages present in starch, maltodextrins, and other related carbohydrates [25]. α-Glucosidase is an enzyme that catalyzes the final step of glucose absorption in the intestine during the digestive process of carbohydrates, hence α-glucosidase inhibitors could retard the rapid hydrolysis of dietary carbohydrates and suppress postprandial hyperglycemia. Dietary starch and other related carbohydrates are digested by α-amylase to a large quantity of maltose, which is further digested by α-glucosidase to glucose to be absorbed in the human intestine [26]. Natural α-amylase and α-glucosidase inhibitors from plant sources offer an attractive strategy to control postprandial hyperglycemia. Especially these, which have a lower inhibitory activity against α-amylase and a stronger inhibitory activity against α-glucosidase, can be used as an effective therapy for postprandial hyperglycemia with minimal side effects [27]. This is because the simultaneous inhibition of both enzymes would result in abnormal bacterial fermentation in the colon due to the presence of undigested carbohydrates, which cause unwanted side effects such as abdominal distention, flatulence, meteorism, and possibly diarrhea [28]. Among the *Allium* species evaluated in this study, *A. akaka* and *A. kharputense* fulfill the condition of possessing a high α-glucosidase inhibitory activity with a low inhibition of α-amylase, suggesting a desirable feature for candidates of nutraceuticals and biotherapeutics. Besides, the extracts showed moderate levels of the pancreatic lipase enzyme, which reduces the formation of lipids in the body and provides a desirable feature to the plant-based foods or nutraceuticals [29].

The scientific reports revealed that allicin- and phenolic-rich *Allium* species were weak alpha-amylase [19] inhibitors, but strong alpha-glucosidase [11] inhibitors. Also, it was found that rutin is an effective agent of antilipase activity [27,28,29,30]. Kim et al. [16] and Schmidt et al. [11] found a positive correlation between the level of quercetin and the antioxidant activity and α-glucosidase activity in several extracts obtained from *Allium cepa*. Nishimura et al. [31] found that consumption of quercetin-rich onion had beneficial effects in preventing obesity and improving liver function through low HDL-C and ALT levels in vivo. Compatibly, there is a positive correlation between the levels of quercetin and its glycosides and biological activities in this study, which suggests that quercetin and its glycosides are among the major contributors of the antilipase and antioxidant activities. Kamalakkanan and Price [32] reported that the oral administration of rutin to diabetic rats significantly decreased fasting plasma glucose and glycosylated hemoglobin, and increased insulin, C-peptide, hemoglobin, thiobarbituric acid reactive substances, and lipid hydroperoxide levels, and increased the non-enzymic antioxidants significantly. The authors also found that the treatment of normal rats with rutin did not significantly alter any of the parameters studied. These findings showed that rutin, a glycoside of quercetin, showed a high antihyperglycemic and antioxidant activity in diabetic rats, but not in healthy ones, which suggests a relationship between oxidative stress and metabolic diseases and the targeted therapeutic effect of the rutin compound. The presence of chlorogenic acid [33,34], rutin [34,35], and allicin [36] in plant extracts provided effective antioxidant abilities, which suggests they are major contributors of antioxidant abilities in *Allium* species grown in Eastern Anatolia. The lipophilic compounds present in the extracts were fatty acids, which were reported as weak antioxidant agents [37], indicating their limited contribution to antioxidant activity.

Chlorogenic acid is a phenolic compound from the hydroxycinnamic acid family, which possesses many biological activities, and it was reported that chlorogenic acid-rich foods had preventive effects such as the suppression of an increase in body fat and sugar cataracts, and a reduction in glucose absorption and therapeutic effects, including improved fasting glucose, reduction in weight, acceleration of diabetic wound healing, and improved symptoms of diseases over metabolic syndromes [38]. Allicin, which is considered a specific representative compound of *Allium* species [39], possesses broad antimicrobial and health-enhancing properties, which ensure they are natural and safe agents against synthetic preservatives and enzyme inhibitory compounds [23].

Due to a higher affinity to specific amino acids of the enzyme’s protein, phenolic compounds can suppress the activity of the enzymes related to metabolic diseases [40]. The phytochemicals present in the extracts were among the most effective enzyme inhibitors. For example, quercetin bound to Asp^80^, Arg^257^, Val^260^, and Ala^261^ amino acids in pancreatic lipase. The size and structural rigidity of quercetin could be responsible for its binding characteristics (more polar interactions) and, consequently, its higher pancreatic lipase inhibitory potency [41].

The antioxidant and enzyme inhibitory activities of quercetin and its glycosides are mainly based on their structural features, which can be summarized as the presence of (i) a catechol structure in the B ring, (ii) a double bond between C2 and C3 in the C ring, and (iii) the number of aromatic and hydroxyl groups. According to the abovementioned criteria, quercetin is one of the most effective antioxidant and enzyme inhibitory compounds among the flavonoids [41], which explains the positive correlation between biological activities and the levels of quercetin and its glycosides (rutin and isoquercitrin) in *Allium* species.

The *Allium* species analyzed within this study had high antioxidant and moderate enzyme inhibitory (alpha-glucosidase and pancreatic lipase) activities and contained phenolic compounds (chlorogenic acid, hesperidin, rutin, isoquercitrin, and quercetin) and allicin as major chemical compounds. These data were similar to previous reports regarding the chemical composition and biological activities of common *Allium* species and can contribute to the scientific database of *Allium* species.

## 4. Materials and Methods

### 4.1. Plant Materials

The leaves of four wild-edible *Allium* species (Table 5) were collected from the highlands of Eastern Anatolia, Turkey on 12 May 2018. The scientific identification was performed at the Van Pharmaceutical Herbarium (VPH), Pharmacy Faculty, Van Yuzuncu Yil University (Van, Turkey). The plant materials were air-dried in the dark at room temperature (22 ± 2 °C) for 96 h. Then, they were ground into a fine powder using a grinding mill (Isolab laboratory mill 602, Interlab, İstanbul, Turkey) and stored at −20 °C for a maximum of 4 weeks until analyzed.

### 4.2. Chemicals

Unless otherwise stated, all chemicals were purchased from Sigma-Aldrich, Inc. (St. Louis, MO, USA) and were of analytical or HPLC grade.

### 4.3. Preparation of Samples for Analysis

#### 4.3.1. Ethanol Extract

The ethanol extracts were prepared as described previously [42]. Briefly, fine powders of the plant materials were mixed with a 10-fold volume of ethanol (80%, obtained from 95%), and shaken for 2 h at room temperature at 15,300× *g* (10,000 rpm in a Beckman JA14 (137 mm) rotor, Sorvall RC-5B centrifuge, Wilmington, DE, USA) at 4 °C for 20 min with the supernatant collected. Subsequently, the supernatants were evaporated under a reduced pressure at 37 °C using a rotary evaporator (Rotavapor R-205, Buchi, Flawil, Switzerland) and then freeze-dried using a lyophilizator (Alpha 1-2 LDplus, Christ, Osterode am Harz, Germany) under a vacuum at −51 °C to fine lyophilized powders. The lyophilized extracts were stored at −20 °C for a maximum of 4 week until analyzed.

#### 4.3.2. Herbal Infusion Extract

The lyophilized herbal infusions were prepared according to Baytop [43]. Briefly, the plant materials were mixed with a 10-fold volume (g/mL) of pre-boiled distilled water and incubated for 10 min. Then, the mixtures were filtered using cotton and vacuum filtering. The filtrates were evaporated and freeze-dried as described previously.

### 4.4. Antioxidant Capacity

#### 4.4.1. Folin-Ciocalteu Reducing (FCR)

The FCR levels of the extracts were measured as described previously [44]. Briefly, 25 µL of the extracts were mixed with 125 µL Folin–Ciocalteu reagent in 96-well microplates and shaken for 3 min. The absorbance was measured at 600 nm for ascorbic acid correction. Subsequently, 125 µL of 6% Na_2_CO_3_ were added and the microplate was shaken for 12 min. The absorbance was then measured at 600 nm. The results were expressed as mg gallic acid (GA) E/g dw based on a GA standard curve. The analyses were conducted in triplicate.

#### 4.4.2. Ferric Reducing Antioxidant Power (FRAP)

The FRAP values of the extracts were determined as described previously [44]. Briefly, 10 µL of the extracts were placed in 96-well microplate and 200 µL of the FRAP reagent was added, followed by 15 s of shaking. After incubation for 8 min, the absorbance was measured at 595 nm using a spectrophotometer and the reducing capacities of the extracts were expressed as mmol of iron (Fe^2+^)/g dw based on an iron sulphate standard (Fe_2_SO_4_) curve against a blank control. The analyses were performed in triplicate.

#### 4.4.3. Oxygen Radical Absorbance Capacity (ORAC)

The ORAC assay was carried out as previously described [44]. Briefly, 20 µL of the extracts were added to a microplate with a 20 min incubation at 37 °C. Subsequently, 20 µL AAPH was added to each well and the fluorescence measurements initiated immediately. Fluorescence (excitation wavelength 495 nm and emission wavelength 515 nm) was measured every min until the fluorescence reached zero and a kinetic curve was obtained using the Mars data analysis software 5.0 (POLARstar Omega, BMG Labtech, Offenburg, Germany). The area under the curve (AUC) was integrated and standardized against a blank control (without AAPH). The measurements were carried out in triplicate. The ORAC levels of the samples were expressed as mmol Trolox equivalents (E)/g dw based on a Trolox standard curve.

### 4.5. Enzyme Inhibitory Activities

#### 4.5.1. α-Glucosidase Inhibitory Activity

The inhibition of the α-glucosidase enzyme was determined as described previously [42]. Briefly, 20 µL of the sample solutions were mixed with 20 µL of 2% sucrose (*w*/*v*) in a maleate buffer. The enzymatic reaction was initiated by adding 20 µL of enzyme solution prepared in 1 mL of 100 mmol maleate buffer and the mixture was incubated at 37 °C for 60 min. The enzymatic reaction was terminated by heating the mixture at 100 °C for 10 min and the absorbance was measured at 505 nm. The relative α-glucosidase inhibition was calculated using the formula: % Inhibition = [((ACB − AC) − (ASB − AS))/(ACB − AC)] × 100, where AS and AC were the absorbance of the sample and the negative control, and where ASB and ACB were the absorbance of the sample blank and the control blank, respectively. Acarbose was used as a positive control.

#### 4.5.2. α-Amylase Inhibitory Activity

The inhibition of α-amylase was performed using the Caraway–Somogyi iodine/potassium iodide (IKI) method as described previously [42]. The assay was conducted with α-amylase Type 1-A: DFP treated from porcine pancreas (Sigma Aldrich, Istanbul, Turkiye) and the Amylase-Test Wako kit (Wako Pure Chemical Institute, Tokyo, Japan).

A sample of starch (200 μL, 0.4 mg/mL) and an extract (100 μL, 20 mg/mL of lyophilized powder) in the phosphate buffer (20 mmol NaH_2_PO_4_ and 6.7 mmol NaCl, pH 6.9) were added to a clean cuvette at room temperature. Subsequently, 50 μL of pancreatic porcine α-amylase solution (1 U/1 mL in the phosphate buffer) was added to the sample mixture, after which the phosphate buffer was added to obtain a final volume of 500 μL. The mixture was incubated at room temperature for 3 min to allow the enzymatic reaction to occur. The reaction was terminated by adding hydrochloric acid (1 mL, 0.1%). Subsequently, an iodine reagent (Wako amylase kit) was added to the mixture. The decrease in starch concentration due to the activity of α-amylase was measured at 660 nm using a Shimadzu 1601 spectrophotometer (Tokyo, Japan). The enzyme inhibitory activities of the extracts were calculated as a percentage of the control using the following equation:

*% Inhibition* = (ACB − AC) − (ASB − AS)/(ACB − AC) × 100

where ACB is the absorbance of the control blank, AC is the absorbance of the control, ASB is the absorbance of the sample blank, and AS is the absorbance of the sample. Acarbose was used as a positive control.

#### 4.5.3. Pancreatic Lipase Inhibitory Activity

The lipase inhibitory activity was assayed as described previously [42]. A total of 25 µL of sample solutions prepared using a citric/phosphate buffer (1 mL of 100 mmol), 50 µL of 4-Muo, and 25 µL of the enzyme solution were placed in a 96-well microplate, inserted into the microplate reader and incubated for 20 min at 37 °C. Subsequently, the reaction was terminated by adding 50 µL of 1 mol hydrochloric acid and 100 µL of sodium citrate, respectively. The relative lipase inhibition activity was calculated using the formula: % Inhibition = (1 − (FS − FSB)/(FC − FCB)) × 100, where FS and FC were the values of the samples and the negative control measured fluorometrically at an emission wavelength of 460 nm and an excitation of 320 nm with slit widths of 5 nm using the microplate reader, and where FSB and FCB were the fluorescence readings of the sample blank and the control blank, respectively. Orlistat was used as positive control.

### 4.6. Phytochemical Profile

#### 4.6.1. Identification and Quantification of Phenolic Compounds

The identification and quantification of phenolic compounds using a high performance liquid chromatography-diode array detector (HPLC-DAD) and liquid chromatography-photo-diode array–mass spectrometry (LC-DAD–MS/MS) on a Quantum triple-stage quadrupole mass spectrometer, equipped with a quaternary solvent delivery system, a column oven, a photo-diode array detector, and an auto sampler (Thermo Fisher Scientific, Waltham, MA, USA), were performed as described previously [42]. An aliquot (3 μL) of each sample solution was chromatographed on a 150 × 2.1 mm, 5 μm Luna Synergy Hydro column (Phenomenex, Torrance, CA, USA), which was heated to 30 °C. The analytes were separated using 0.5% formic acid and acetonitrile (200 µL/min flow rate). The photodiode array detector was used to obtain data from 190–520 nm. The composition of phenolic compounds was identified based on their UV spectrum, retention time, co-chromatography with commercial standards, when available, and MS/MS fragmentation patterns. Mass spectrometry data were obtained using an electrospray source in either the positive (quercetin 3-glucoside) or the negative (chlorogenic acid) modes. MS experiments in the full scan (parent and product-specific) and the selected reaction monitoring (SRM) mode were performed. The quantification of phenolic compounds was performed using the HPLC-DAD system, which consisted of two LC-10ADVP pumps, an SPD-M10ADVP diode array detector, a CTO-1-ADVP column oven, a DGU-12A degasser, an SIL-10ADVP auto injector, and an SCL-10A system controller equipped with an Atlantis column (dC_18_, 4.6 × 100 mm, 5 µm particle size) (Waters Associates, Milford, MA, USA). Analytical HPLC was run at 30 °C and monitored at 250, 280, 320, 370, and 520 nm. The injection volume was 10 µL. The levels of the phenolic compounds were quantified as an authentic standard E/g dw based on a calibration curve.

#### 4.6.2. Identification and Quantification of Fatty Acid (FA) Compounds

The FA compounds present in lyophilized extracts were measured using gas chromatography mass spectrometry (GC/MS) (3800 Varian GC, Agilent Technologies, Istanbul, Turkey) using a headspace solid-phase microextraction as described previously by Uzun et al. [45]. Briefly, the extracts were chromatographed on a 60 × 0.25 mm i.d., 0.2 μm HP 88 column (Agilent Technologies, Istanbul, Turkey). The oven temperature was programmed as follows: 80 °C at the start, followed by an increase to 190 °C at a rate of 5 °C/min, held at 190 °C for 3 min, followed by an increase at a rate of 5 °C/min to 260 °C, and held for 3 min. The subsequent temperature increase was at a rate of 2 °C/min to 275 °C and held for 12.5 min. The carrier gas was helium, used at a constant pressure of 10 psi; the transfer line temperature was 250 °C; electron impact (EI); acquisition ion range, 40 to 200 m/z; scan rate.

### 4.7. Data Analysis

A one-way ANOVA with the Bonferroni post hoc test was used to measure differences between the samples at *p* < 0.05 using Graphpad Prism 5 (Graphpad Software, San Diego, CA, USA). All the IC_50_ values were determined through the corresponding dose-inhibition curves according to their best-fit shapes.

## 5. Conclusions

This is the first report of the biological activities and phytochemical composition of *Allium* species, including *A. akaka*, *A. affine*, *A. kharputense*, and *A. shirnakiense*, grown and consumed as food in daily life in the highlands of Eastern Anatolia. Th phytochemical compounds detected within this study (allicin and various phenolics) were among the major contributors to the antioxidant and enzyme inhibitory activities, with the contribution of fatty acids. Catechol moiety-containing compounds rich in ethanol-based leaf extracts had a superior antioxidant capacity both for the HAT and SET mechanisms and the inhibition of of α-glucosidase and pancreatic lipase. Ethanol extracts and infusion preparations of *Allium akaka* and *Allium kharputense* were found to be potent sources of functional foods and/or biotherapeutics, which contain significant phytochemicals.

## Figures and Tables

**Table 1:** Yields, total phenolic contents, and antioxidant activities.

	Extract	*A. kharputense*	*A. affine*	*A. shirnakiense*	*A. akaka*
**Yield (%)**	Ethanol	12.5 ± 1	17.5 ± 1	11.3 ± 0	18.2 ± 1
	Infusion	16.8 ± 1	19.4 ± 2	14.8 ± 1	20.1 ± 1
**Total Phenolic Content** **(mg gallic acid E/g extract)**	Ethanol	43 ± 1b	25.8 ± 1.4d	33 ± 2c	48 ± 3a
	Infusion	39 ± 2a	18 ± 1d	25 ± 1c	32 ± 1b
**FRAP** **(μmol Fe^2+^/g extract)**	Ethanol	836 ± 62a	469 ± 10c	572 ± 17b	872 ± 5a
	Infusion	640 ± 28a	318 ± 5d	384 ± 10c	545 ± 8b
**ORAC** **(μmol Trolox/g extract)**	Ethanol	1835 ± 46a	1133 ± 12c	1606 ± 29b	1858 ± 1a
	Infusion	1141 ± 30a	723 ± 37b	1185 ± 69a	1115 ± 24a

Means with different letters in the same row were significantly different at the level (*p* < 0.05); n = 3.

**Table 2:** Enzyme inhibitory activities.

**α-Amylase (IC-50; mg/mL)**					
	**Extract**	***A. kharputense***	***A. affine***	***A. shirnakiense***	***A. akaka***
	Ethanol	6.2 ± 0.5b	4.5 ± 0.7a	4.8 ± 0.2a	6.5 ± 0.4b
	Infusion	5.0 ± 0.3b	4.0 ± 0.4a	4.2 ± 0.1a	4.7 ± 0.3b
Positive control (Acarbose)		0.22 ± 0.03			
**α-Glucosidase (IC-50; mg/mL)**					
	Ethanol	0.6 ± 0.1b	0.9 ± 0.1d	0.9 ± 0.0c	0.5 ± 0.1a
	Infusion	0.7 ± 0.1a	1.0 ± 0.0d	1.0 ± 0.0c	0.9 ± 0.0b
Positive control (Acarbose)		0.081 ± 0.00			
**Pancreatic lipase (IC-50; µg/mL)**					
	Ethanol	218 ± 8b	413 ± 15d	324 ± 13c	195 ± 10a
	Infusion	258 ± 9a	549 ± 12d	452 ± 19c	347 ± 10b
Positive control (Orlistat)		76 ± 1.4			

Means with different letters in the same row were significantly different at the level (*p* < 0.05); n = 3.

**Table 3:** Phytochemical composition using HPLC-MS/MS.

Compound	MS/MS		Concentration (mg/g Extract)							
			*A. kharputense*		*A. affine*		*A. shirnakiense*		*A. akaka*	
			Leaf		Leaf		Leaf		Leaf	
	[M + 1]^+^/[M − 1]	Fragments (m/z) (+/−)	Ethanol	Infusion	Ethanol	Infusion	Ethanol	Infusion	Ethanol	Infusion
***Chlorogenic acid***	-/353	-/191	3.6 ± 0.1	3.9 ± 0.0	1.8 ± 0.0	1.2 ± 0.0	0.7 ± 0.0	0.6 ± 0.0	6.6 ± 0.4	2.4 ± 0.2
***Hesperidin***	/609	/449,431	3.1 ± 0.0	3.2 ± 0.0	2.1 ± 0.0	1.9 ± 0.0	3.5 ± 0.0	3.0 ± 0.0	4.1 ± 0.2	3.9 ± 0.1
***Rutin***	611/609	303/301	5.7 ± 0.2	4.2 ± 0.1	13.8 ± 0.2	4.2 ± 0.1	3.5 ± 0.1	6.8 ± 0.4	25 ± 1	13 ± 1
***Isoquercitrin***	-/463	-/301	13.2 ± 0.2	7.8 ± 0.3	3.6 ± 0.2	2.4 ± 0.1	10.7 ± 0.6	4.5 ± 0.2	8.4 ± 0.2	4.8 ± 0.2
***Quercetin***	-/301	-/227, 151	2.4 ± 0.0	1.5 ± 0.0	9.1 ± 0.5	1.7 ± 0.1	3.1 ± 0.1	1.3 ± 0.1	5.4 ± 0.2	0.8 ± 0.0
***Allicin***	163/	121/-	7.6 ± 0.0	7.4 ± 0.3	8.0 ± 0.1	7.3 ± 0.1	7.3 ± 0.1	7.0 ± 0.2	7.0 ± 0.2	6.1 ± 0.2

ND: not detected; T: traces.

**Table 4:** Fatty acid composition using GC-MS.

Compound	Retention Time	Concentration (%)							
		*A. kharputense*		*A. affine*		*A. shirnakiense*		*A. akaka*	
		Leaf		Leaf		Leaf		Leaf	
		Ethanol	Infusion	Ethanol	Infusion	Ethanol	Infusion	Ethanol	Infusion
**Palmitic acid**	36.8	23.7 ± 2	51.3 ± 3	34.7 ± 3	34.3 ± 2	24.8 ± 2	100 ± 0	26.6 ± 1	24.9 ± 1
**Stearic acid**	40.1	3.2 ± 0	33.0 ± 2	6.0 ± 0	42.4 ± 3	2.6 ± 0	ND	3.4 ± 0	ND
**Oleic acid**	40.8	4.1 ± 0	ND	8.4 ± 1	ND	2.5 ± 0	ND	ND	ND
**Linoleic acid**	42.2	18.7 ± 1	T	32.3 ± 2	14.0 ± 1	13.8 ± 1	ND	15.6 ± 1	21.0 ± 2
**Linolenic acid**	43.6	42.6 ± 3	15.6 ± 1	18.3 ± 1	T	52.4 ± 3	ND	54.2 ± 4	53.9 ± 3

ND: not detected; T: traces.

**Table 5:** Botanical and ethnobotanical knowledge of wild-edible *Allium* taxa from the highlands of Eastern Anatolia.

	*A. kharputense* Freyn & Sint.	*A. affine* Ledeb.	*A. shirnakiense* L.Behçet & Rüstemoğlu	*A. akaka* S.G.Gmel.
Location	Konalga village, Çatak, Van/Turkey on 12 May 2018 (Global Positioning System (GPS) coordinates: 37°50′1915″ N 43°08′1851″ E, 2454 m)	Konalga village, Çatak, Van/Turkey on 12 May 2018 (GPS coordinates: 37°50′1915″ N 43°08′1851″ E, 2454 m)	Konalga village, Çatak, Van/Turkey on 12 May 2018 (GPS coordinates: 37°50′1915″ N 43°08′1851″ E, 2454 m)	Konalga village, Çatak, Van/Turkey on 12 May 2018 (GPS coordinates: 37°50′1915″ N 43°08′1851″ E, 2454 m)
Local names	Soryaz	Pîvaza se	Guhbizin	Guhbizin
Collector code	MM301	MM337	MM768	MM767
Herbarium code	VPH338	VPH314	VPH329	VPH351
Part used	Leaves	Leaves	Leaves	Leaves
Medicinal use	Diabetes	Diabetes	Diabetes	Diabetes
Medicinal utilization	Eaten fresh or infusion	Eaten fresh or infusion	Eaten fresh or infusion	Eaten fresh or infusion
Food utilization	Eaten fresh or omelet with eggs	Eaten fresh or omelet with eggs	Eaten fresh or omelet with eggs	Eaten fresh or omelet with eggs
Growth and developmental properties during harvest	Bulbs ovoid and 2–3 cm diam. Stem 30–50 cm long. 2–3 leaves on each plant, leaves broadly lanceolate-shaped, 18–35 mm broad, and aculeolate at margin. Spathe 2–3-lobed. Umbel many-flowered and 4–8 cm diam. Pedicels slender-shaped and straw-colored. Perianth segments cream, linear-shaped, 5–6 mm long, and somewhat reflexed. Filaments lanceolate base, as long as or longer than perianth. Anthers cream.	Bulb ovoid-shaped and 1–2 cm diam. Stem 15–40 cm long. Leaves 3–4 pieces, 3–4 mm broad, semicylindrical, canaliculate. Spathe 1 and valved. Umbel many-flowered, spherical and 2–3 cm diam. Pedicels unequal, slender-shaped, thickened base. Perianth segments whitish, linea; perianth narrowly campanulate-shaped.	Bulb globose and 3–3.5 × 3.5–4.5 cm diam. Stem 20–35 cm long, erect, and longer than leaves. Leaves ligulate-shape, plant 1–4 leaves, 12–20 cm long, and 4–7 cm wide. Spathe 3–4 valved, valves ovate; spathe shorter than umbel. Umbel globose-shaped, many flowered 5 cm diam. Pedicels to 23 mm long, greenish-purple. Perigon stellate with tepals brownish green, lanceolate, Perianth segments linear-lanceolate. Filaments included brown, purplish-brown. Anthers 2–2.5 mm long, slightly exserted, pale brown or greenish-brown.	Bulbs 2–3 cm diam. Leaves 1–3, elliptic-oblong 2–6 cm broad, glaucous, obtuse, and mucronate, scabrid on margin. Spathe is 1–2 cm long, has 2–4 lobes, and shorter than an umbrella. Umbrella with many flowers and erect, parallel branches that are hemispherical and measure 5–9 cm in diameter. Pedicels 2–3 cm long. Perianth campanulate-shaped. Perianth segments purplish-pink, narrowly elliptic-oblong and 7–8 mm lond. Filaments pinkish-purple.
Wild photo	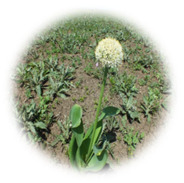	* 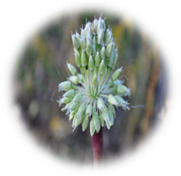 *	* 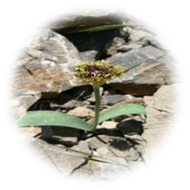 *	* 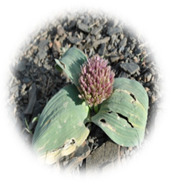 *

## Data Availability

The original contributions presented in the study are included in the article, further inquiries can be directed to the corresponding author.
